# Innate lymphoid cell (ILC) subsets are enriched in the skin of patients with hidradenitis suppurativa

**DOI:** 10.1371/journal.pone.0281688

**Published:** 2023-02-13

**Authors:** Andreea Petrasca, Roisin Hambly, Oonagh Molloy, Sean Kearns, Barry Moran, Conor M. Smith, Rosalind Hughes, Margaret O’Donnell, Alexandra Zaborowski, Desmond Winter, Jean M. Fletcher, Brian Kirby, Anna Malara

**Affiliations:** 1 School of Biochemistry and Immunology, Trinity Biomedical Sciences Institute, Trinity College Dublin, Dublin, Ireland; 2 Department of Dermatology, St. Vincent’s University Hospital, Dublin, Ireland; 3 School of Medicine, University College Dublin, Dublin, Ireland; 4 Plastic and Reconstructive Surgery Department, St. Vincent’s Private Hospital, Dublin, Ireland; 5 Department of Surgery, St. Vincent’s University Hospital, Dublin, Ireland; 6 School of Medicine, Trinity Biomedical Sciences Institute, Trinity College Dublin, Dublin, Ireland; The University of Texas MD Anderson Cancer Center, UNITED STATES

## Abstract

Hidradenitis suppurativa (HS) is a chronic relapsing inflammatory skin disease manifested as painful inflamed lesions including deep-seated nodules, abscesses and sinus tracts. The exact aetiology of HS is unclear. Recent evidence suggests that immune dysregulation plays a crucial role in pathogenesis and disease progression. Innate lymphoid cells (ILC) are a recently identified immune cell subset involved in mediating immunity, however their role in HS has not yet been investigated. Three distinct subsets of ILC- ILC1, ILC2 and ILC3 have been described, and these are involved in skin tissue homeostasis and pathologic inflammation associated with autoimmunity and allergic diseases. In this study, we analysed by multiparameter flow cytometry the frequencies of ILC subsets in skin and peripheral blood mononuclear cells (PBMC) of HS patients and compared these to healthy control subjects and psoriasis patients. The absolute numbers of total ILC and subsets thereof were significantly reduced in the blood of HS patients relative to healthy controls. However, when patients were stratified according to treatment, this reduction was no longer observed in patients undergoing anti-TNF treatment. In HS lesional skin the absolute numbers of ILC were significantly increased relative to control skin. Furthermore, the frequencies of total ILC as well as ILC2 and ILC3 were significantly higher in non-lesional than lesional HS skin. This study analysed for the first time the presence of ILC subsets in the blood and skin of HS patients. Our findings suggest that ILC may participate in HS pathogenesis.

## Introduction

Hidradenitis suppurativa (HS), is a chronic relapsing inflammatory skin disease affecting the axillae, perineum and inframammary region. HS is characterised by initial occlusion of hair follicles, caused by keratosis and hyperplasia of the follicular epithelium, followed by follicular rupture and subsequent acute inflammation. This results in the formation of painful subcutaneous nodules and abscesses, as diseases progresses, formation of sinus tracts and scarring [1]. HS is associated with a significant social and psychological morbidity. The prevalence of HS is estimated to range from 0.05% to 4% [2].

The exact aetiology of HS is unknown, however, there is clear evidence indicating that immune dysregulation is a key factor responsible for disease pathogenesis and progression. Previous studies demonstrated elevated levels of Th17 cell-associated cytokines including IL-1, IL-23 and IL-17, but also increased TNF-α and IL-10 in HS skin [3–5]. Further studies showed increased infiltration of polyfunctional Th17 cells and dysregulation of Th17/Treg ratio in HS skin lesions compared to uninvolved skin [6]. Elevated levels of IL-17, TNF-α and IL-10 in the serum have also been observed in HS patients [4, 7, 8]. Moreover, the lesional skin of HS patients exhibits an enrichment of B cells and plasma cells [9, 10].

Innate lymphoid cells (ILC) are tissue resident hematopoietic cells of the lymphoid lineage, which do not express antigen-specific receptors that are typically found in T cells and B cells. ILC, which respond directly to innate signals and cytokines [11, 12], are divided into subpopulations based on their cytokine expression profile and they are the innate equivalent of T helper cell subsets. Human ILC group 1 (ILC1) produce Th1 cytokines IFN-γ and TNF-α, while ILC group 2 (ILC2) produce Th2 cytokines IL-13 and IL-5, and ILC group 3 (ILC3) produce Th17 cytokines IL-17 and IL-22 [13]. Human ILC subsets can be identified by expression of cell surface markers. ILC do not express cell lineage markers characteristic to T cells, B cells, dendritic cells, macrophages and granulocytes (Lin^-^), but express IL7Rα (CD127^+^). ILC2 are further characterised by expression of chemoattractant receptor-homologous molecule (CRTH2, CD294), also known as prostaglandin D2 receptor 2 and expressed on Th2 cells. ILC3 express CD117 (c-Kit; SCFR, mast/stem cell growth factor receptor) and can be subdivided based on expression of natural cytotoxicity receptors (NCR) into ILC3 NKp44^+^, and ILC3 NKp44^-^. A subpopulation of ILC3 expressing NKp46^+^ has been also identified. Human ILC1 do not express CD117 or NKp44 and mostly express CD161 [14, 15]. ILC play important roles in mediating tissue homeostasis and immunity but they have also been implicated in inflammatory skin disorders [13]. Recent reports identified increased levels of IL-22-producing ILC3 in blood and skin of psoriasis patients [16–18], whereas ILC2 were found to be increased in skin and blood of patients with atopic dermatitis (AD) [19, 20]. To date, ILC subsets have not been investigated in HS. Therefore, in this study, we characterised the frequencies of ILC in the skin and blood of patients with HS compared to healthy control volunteers and psoriasis patients.

## Materials and methods

### Patient recruitment

Healthy, HS and psoriasis blood was attained from patients recruited at dermatology clinics at St Vincent’s University Hospital, Dublin. HS patients undergoing surgical procedures at St Michael’s Hospital, Dublin were recruited to donate skin biopsies. The 6-mm punch biopsies were obtained from the leading edge of an active nodule (lesional sample) and from normal-appearing uninvolved skin 10 cm from the active lesion (non-lesional). Healthy skin, which would have otherwise been discarded, was obtained from patients undergoing procedures at St Vincent’s Private Hospital Plastic and Reconstructive Surgery Department. The study received ethical approval from the Medical Research Ethics Committee of St Vincent’s University Hospital. The study received ethical approval from the Research Ethics Committee of St Vincent’s University Hospital, reference number: RS18-026 and REG19-00. A written informed consent was obtained from healthy volunteers, HS and psoriasis patients.

### Cell isolation and staining for flow cytometry

**Fig 1:**
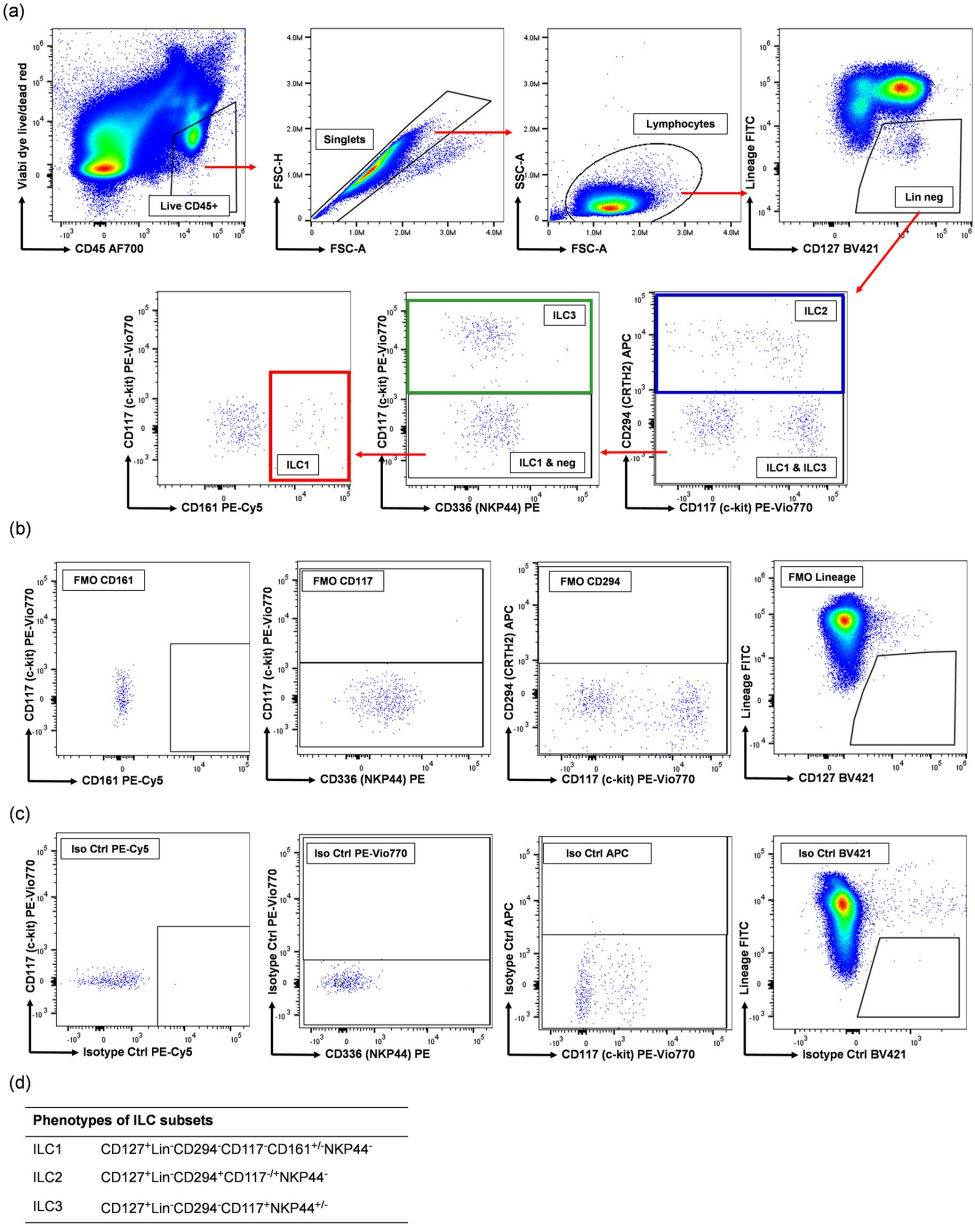
Flow cytometric gating strategy for ILC1, ILC2 and ILC3. ILC were defined as live CD45^+^ cells, which were then gated on forward scatter height (FSC-H) vs FSC area (FSC-A) to exclude doublets. Singlets were than gated on side scatter area (SSC-A) vs FSC-A to select lymphocytes. ILC were then defined as CD127^+^ lineage-negative, with the lineage ‘cocktail’ consisting of antibodies specific for CD1a, CD3, CD11c, CD14, CD19, CD34, CD94, CD123, CD303, FCεRI, TCRαβ and TCRγδ. ILC1 were then defined as CD117^-^CD161^+^, ILC2 as CD294^+^ and ILC3 as CD117^+^ NKP44^+/-^ (a). Cell populations were identified based on Fluorescence Minus One (FMO) controls (b) and isotype controls (c). Phenotypes of ILC subsets are described in table in panel (d).

Peripheral blood mononuclear cells (PBMC) were isolated from fresh blood by density gradient centrifugation over Lymphoprep (Fresenius Kabi, Norway) and cryopreserved. Whole Skin Dissociation Kit (Miltenyi Biotec, Germany) was used to obtain a single-cell suspension from skin tissue. PBMC or freshly-isolated skin cells were treated with Human TruStain FcX^™^ Fc receptor blocking solution (BioLegend, USA) and prepared for flow cytometric analysis by staining with LIVE/DEAD^™^ Fixable Red Dead Cell Stain (Invitrogen, USA) and fluorochrome-conjugated monoclonal antibodies specific for CD1a, CD3, CD11c, CD14, CD19, CD34, CD94, CD123, CD303, FCεRI, TCRαβ, TCRγδ (FITC), CD294 (APC), CD45 (AF700), CD127 (BV421), CD336 (PE), CD161 (PE-Cy5) and CD117 (PE-Vio770) purchased from Thermo Fisher Scientific (USA), BioLegend, BD Biosciences (USA) and Miltenyi Biotec. The cells were subsequently fixed with 1% paraformaldehyde and acquired on a BD LSR Fortessa cytometer (BD Biosciences) within 24 h. Immediately prior to acquisition, Precision Count Beads^™^ (BioLegend) were added to the samples to allow for calculation of cell numbers. Data were analysed using FlowJo software v10 (FlowJo, USA). The different ILC subsets were analysed using the gating strategy outlined in Fig 1.

### Statistical analysis

Statistical analyses were performed using GraphPad Prism v8 software (Graphpad, USA). Mann-Whitney tests were used to compare two groups, while Kruskal-Wallis tests with Dunn’s multiple comparisons were used to compare three groups. Differences of *P* <0.05, *P* <0.01 and *P* <0.001 were considered statistically significant.

## Results

### Frequencies of ILC are increased in non-lesional skin

**Fig 2:**
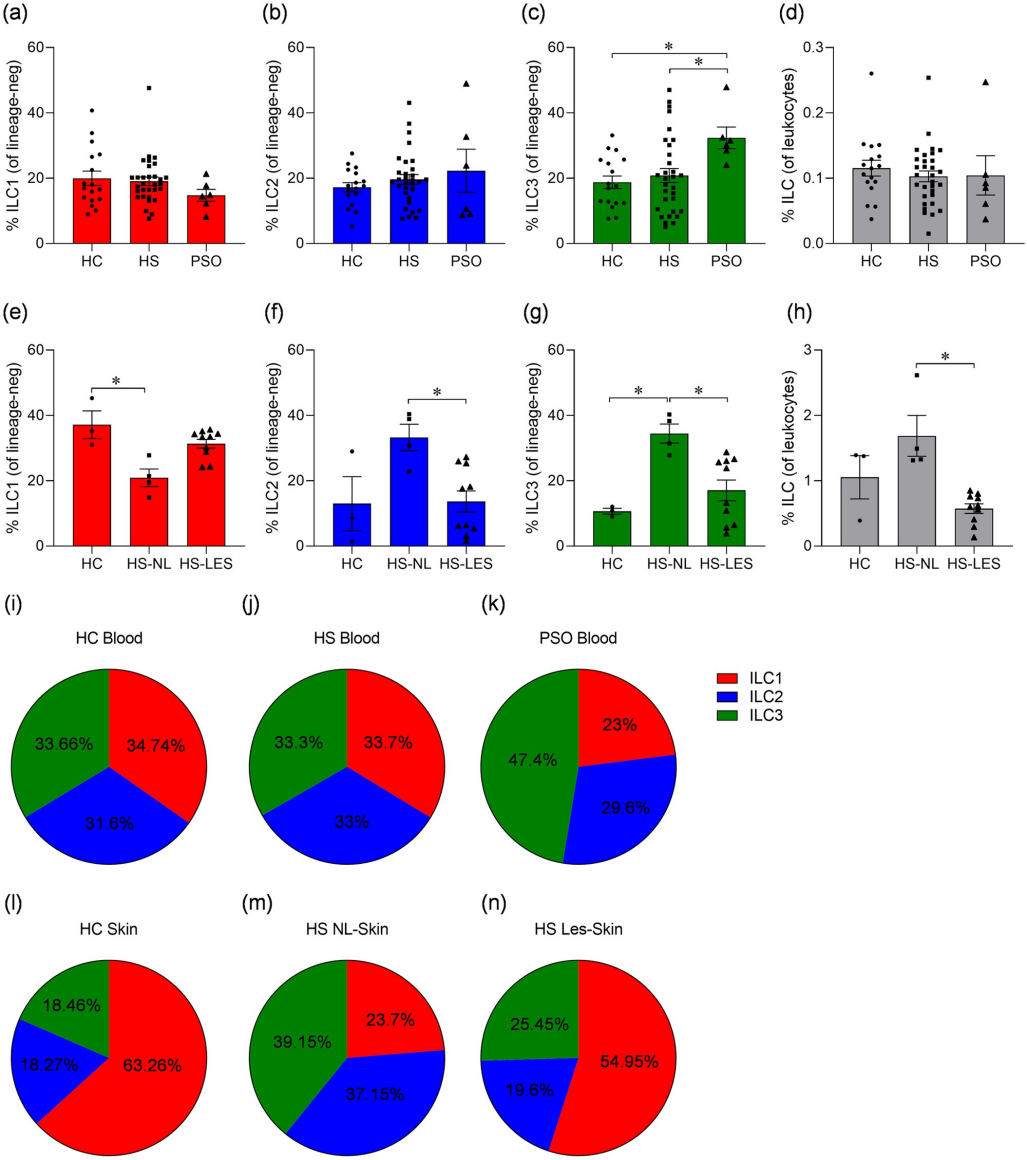
ILC frequencies in the blood and skin of hidradenitis suppurativa (HS) and psoriasis (PSO) patients compared to healthy controls (HC). PBMC and skin-derived cells were analysed by flow cytometry for frequencies of ILC types 1 (ILC1), 2 (ILC2) and 3 (ILC3) and total ILC. ILC were defined as CD45^+^CD127^+^ lineage-negative, with ILC1 defined as CD117^-^CD161^+^, ILC2 defined as CD294^+^ and ILC3 defined as CD117^+^ NKP44^+/-^. Lineage ‘cocktail’ included antibodies specific for CD1a, CD3, CD11c, CD14, CD19, CD34, CD94, CD123, CD303, FCεRI, TCRαβ and TCRγδ. (a-c, e-g) average (± SEM) frequency of ILC1, ILC2, ILC3 (% of lineage-negative cells) in blood of healthy controls (HC, n = 17) and HS (n = 31) or psoriasis (PSO, n = 6)) patients (a-c), and skin of healthy individuals (HC, n = 3) and non-lesional (NL, n = 4) or lesional (LES, n = 10) skin of HS patients (e-g). (d,h) average (± SEM) frequency of total ILC of CD45^+^ cells (leukocytes) in blood (d) and skin (h). (i-n) pie charts depicting frequencies of ILC1, ILC2 and ILC3 as a proportion of total ILC in blood (i-k) and skin (l-n). *P<0.05 using Kruskal-Wallis test with Dunn’s multiple comparisons.

ILC were shown to play a role in regulating local immune responses in the skin [13] and they may also contribute to the pathogenesis of psoriasis [16–18]. In this study we investigated how the frequencies of ILC in HS patients compare to healthy subjects and psoriasis patients. Our results showed no change in the frequencies of ILC subsets in HS compared to healthy control blood, while ILC3 were increased in the blood of psoriasis patients versus healthy controls (p = 0.03, Fig 2a–2d, S1a Fig)., in line with a previous report [17].

The frequency of ILC1 in HS non-lesional skin was significantly decreased compared to healthy control skin (p = 0.017), while percentages of ILC2 (p = 0.048), ILC3 (p = 0.028) and total ILC (p = 0.018) were increased in non-lesional skin compared to lesional skin (Fig 2e–2h, S1b Fig). The frequency of ILC3 in non-lesional skin was also significantly increased compared to healthy control skin (p = 0.039). These changes were also observed when ILC were expressed as a percentage of total ILC (Fig 2i–2n). In summary, while the frequencies of ILC subsets were unchanged in the blood of HS patients relative to healthy controls, there was an increased frequency of total ILC as well as ILC2 and ILC3 in non-lesional versus lesional HS skin.

### ILC numbers are decreased in HS blood, but increased in lesional skin

**Fig 3:**
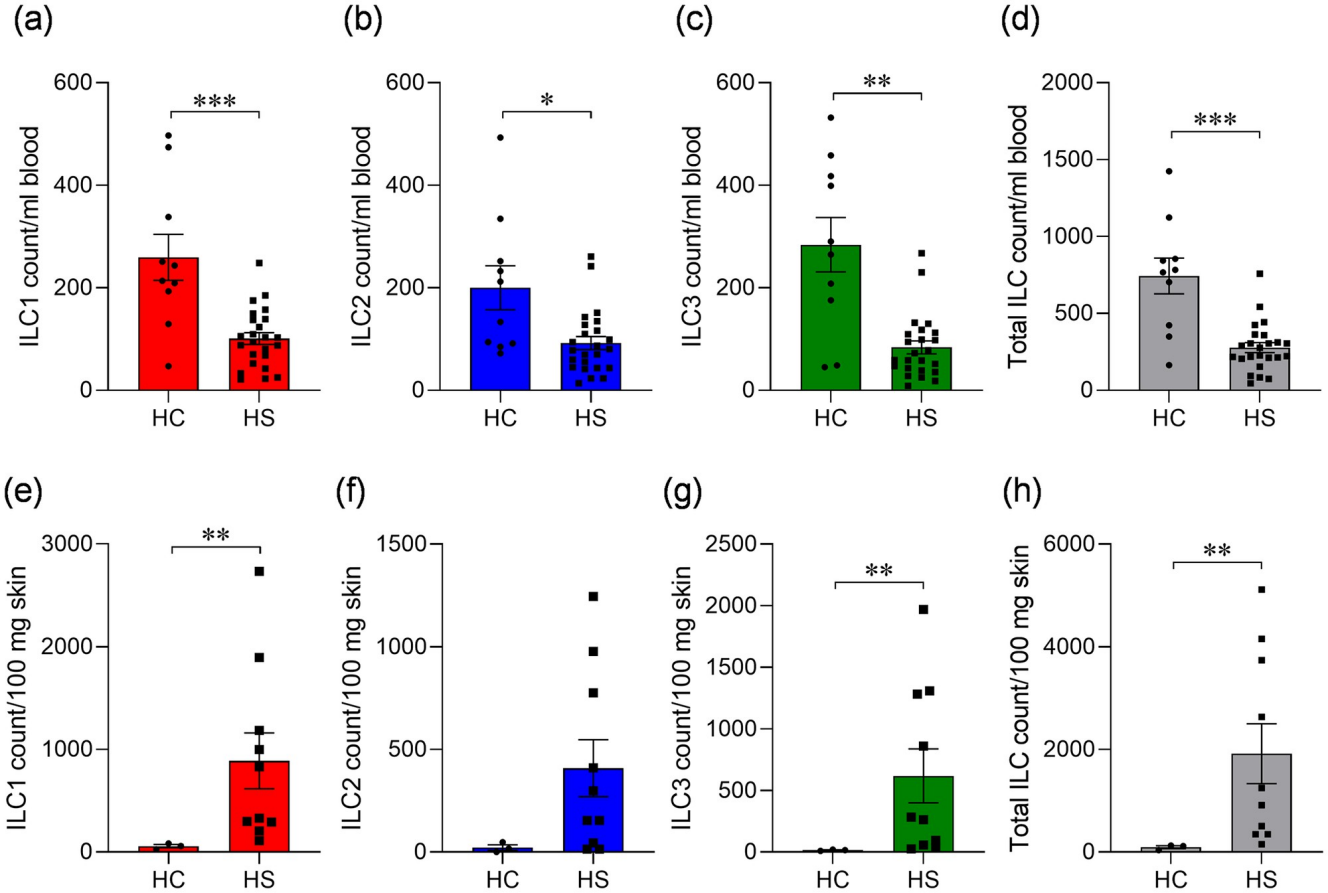
Absolute numbers of ILC in the blood and skin of hidradenitis suppurativa (HS) patients compared to healthy controls (HC). PBMC and skin-derived cells were analysed by flow cytometry for total numbers of ILC types 1 (ILC1), 2 (ILC2) and 3 (ILC3) and total ILC. ILC were defined as CD45^+^CD127^+^ lineage-negative, with ILC1 defined as CD117^-^CD161^+^, ILC2 defined as CD294^+^ and ILC3 defined as CD117^+^ NKP44^+/-^. Lineage ‘cocktail’ included antibodies specific for CD1a, CD3, CD11c, CD14, CD19, CD34, CD94, CD123, CD303, FCεRI, TCRαβ and TCRγδ. Precision count beads were used to calculate cell numbers. (a-h) cell counts (± SEM) of ILC1, ILC2, ILC3 and total ILC per ml of blood in healthy controls (HC, n = 10) and HS (n = 24) patients (a-d), and per 100 mg of skin of healthy individuals (HC, n = 3) and lesional skin of HS patients (n = 10) (e-h). *P<0.05, **P<0.01, ***P<0.001 using Mann-Whitney tests.

Upon examining the absolute counts of ILC in blood of healthy controls, we observed the numbers of ILC in line with previous reports [21–23]. The results showed that the numbers of ILC1 (p = 0.0003), ILC2 (p = 0.0115), ILC3 (p = 0.0013), and total ILC (p = 0.0003) were decreased in the blood of HS patients compared to healthy controls (Fig 3a–3d). In contrast, the numbers of ILC1 (p = 0.007), ILC2, ILC3 (p = 0.007), and total ILC (p = 0.007) were increased in lesional skin of HS patients when compared to healthy control skin (Fig 3e–3h). In summary, ILC appeared to be depleted in the blood and enriched in the skin of HS patients relative to healthy controls.

### ILC counts in the blood are increased in HS patients on anti-TNF treatment compared to those on non-biologics

**Fig 4:**
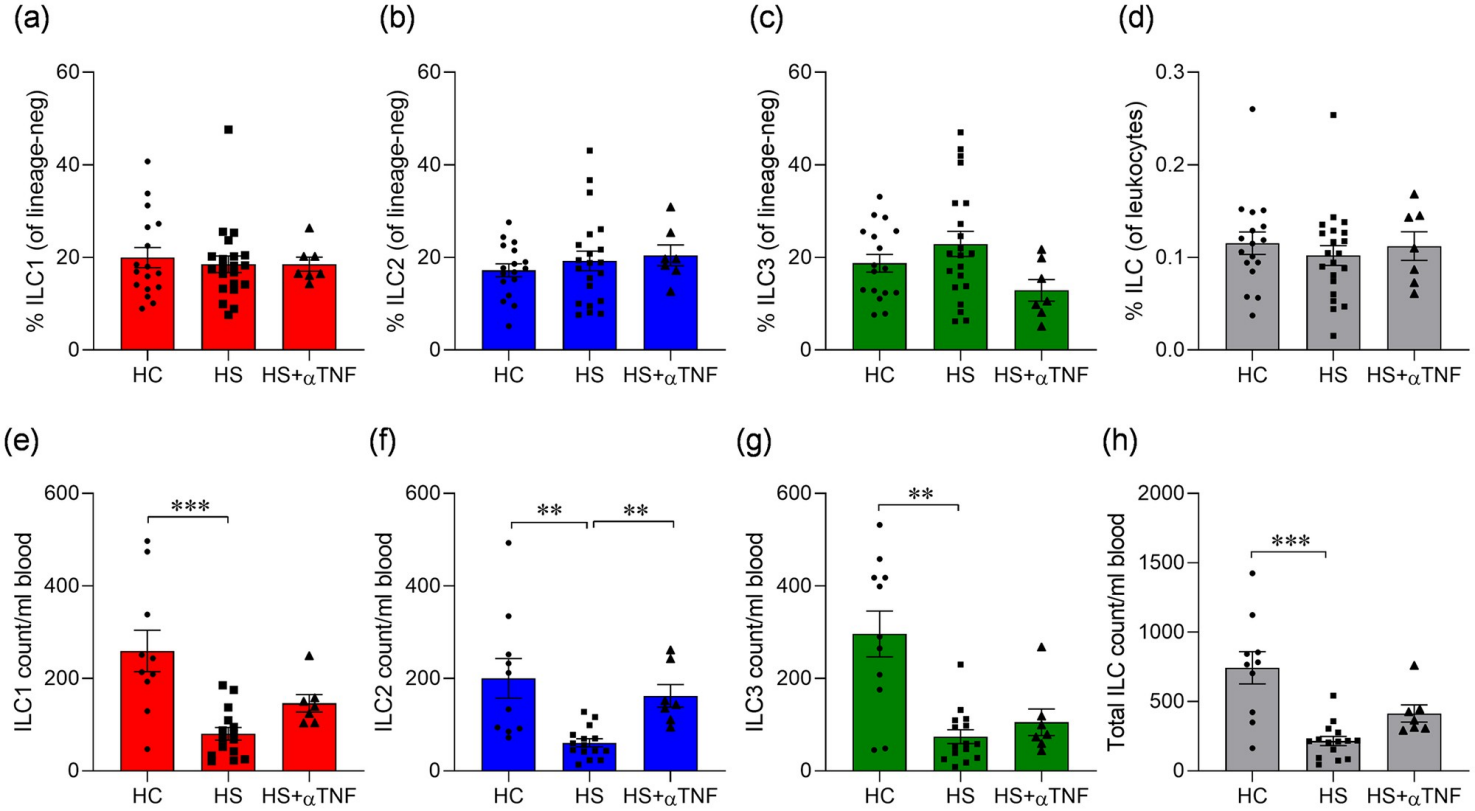
Frequencies and absolute numbers of ILC in the blood of hidradenitis suppurativa (HS) patients on anti-TNF treatment or non-biologics, compared to healthy controls (HC). PBMC were analysed by flow cytometry for frequencies of ILC types 1 (ILC1), 2 (ILC2) and 3 (ILC3) and total ILC. ILC were defined as CD45^+^CD127^+^ lineage-negative, with ILC1 defined as CD117^-^CD161^+^, ILC2 defined as CD294^+^ and ILC3 defined as CD117^+^ NKP44^+/-^. Lineage ‘cocktail’ included antibodies specific for CD1a, CD3, CD11c, CD14, CD19, CD34, CD94, CD123, CD303, FCεRI, TCRαβ and TCRγδ. Precision count beads were used to calculate cell numbers. (a-d) average (± SEM) frequency of ILC1, ILC2 and ILC3 (% of lineage-negative cells (a-c) or total ILC (% of leukocytes) (d) in blood of healthy controls (HC, n = 10), and HS patients on non-biologic therapy (HS, n = 21) or anti-TNF (HS+αTNF, n = 7). (e-h) cell counts (± SEM) of ILC1, ILC2, ILC3 and total ILC per ml of blood in healthy controls (HC, n = 10), and HS patients on non-biologic therapy (HS, n = 15) or anti-TNF (HS+αTNF, n = 7). **P<0.01, ***P<0.001 using Kruskal-Wallis test with Dunn’s multiple comparisons.

The effect of treatment strategy on ILC subsets in HS is not known, therefore, we examined whether patients on anti-TNF treatment exhibited differences in their ILC subsets compared to patients on non-biological treatment. Blood samples from 15 patients on non-biologic therapy and 7 patients, who were treated with anti-TNF were analysed. We found that ILC frequencies were comparable in both patient groups (Fig 4a–4d). However, in patients on anti-TNF therapy, the numbers of total ILC as well as ILC1 and ILC2 (p = 0.002) appeared to be restored to levels similar to those found in healthy controls (Fig 4e–4h).

## Discussion

This study was the first to immunophenotype ILC subsets in PBMC and skin of patients with HS through flow cytometric analysis. We demonstrated that the frequency of CD45^+^Lin^-^CD127^+^ ILC is higher in the non-lesional HS skin compared to lesional skin. Particularly, significantly elevated frequencies of ILC2 and ILC3, but not ILC1 were seen in the non-lesional skin compared to lesional skin of HS patients. We observed enrichment of ILC3 in the non-lesional skin, suggesting that these cells may play a role in early phase of inflammatory response and lesion formation in HS skin. ILC3 have been shown to produce IL-22 and IL-17 in the skin, following activation by IL-1 and IL-23 [18]. Therefore, ILC3 may partly mediate the initial stage of the inflammation in HS skin, which is subsequently replaced and sustained by adaptive Th mediated response, with expression of multiple Th17- and Th1-derived cytokines and followed by recruitment of other immune cells, such as neutrophils, which contribute to formation of severe active lesions. Interestingly, ILC3-like cells, expressing *Rorc* transcription factor and producing TNF-α and lymphotoxins (LT) have been identified in mouse skin. Under homeostatic condition, these ILC cells were found to negatively regulate sebaceous gland function, on the TNF- α and LT-dependent manner and through inhibition of Notch pathway [24]. Impaired Notch signalling has been proposed to be one of the mechanisms of HS pathogenesis [25]. Mutations in Notch signalling pathway have been identified by whole-exome sequencing of patients with HS [26]. Moreover, sebaceous glands were found to be reduced in hair follicles of uninvolved HS skin [27]. These finding suggest that ILC3 could contribute to HS pathogenesis through regulation of Notch signalling.

We also observed an increased frequency of ILC2 cells in the non-lesional HS skin. ILC were reported to be increased in AD skin lesions, and are therefore implicated in AD pathogenesis [19]. Furthermore, ILC2 signature cytokines IL-5 and IL-13 control B cells maturation and differentiation [28]. Recent reports have demonstrated elevated levels of B cells, plasma cells and plasmablasts in HS skin by flow cytometry [9], while single cell RNA sequencing analysis of HS skin samples indicated a critical role of B cell responses in HS pathogenesis [10].

ILC1 have been shown to produce TNF-α, an inflammatory cytokine, which is highly elevated in HS skin and a target for biologic HS treatment, our results found no change in the level of ILC1 between the lesional HS skin and healthy control skin. Moreover, we observed significantly lower frequency of ILC1 in the non-lesional HS skin compared to healthy controls, which suggests that ILC1 might not notably contribute to the initiation of the inflammatory process in HS skin. We observed that the proportion of ILC subsets in the lesional HS skin is similar to the proportion of ILC subsets in healthy control skin, with ILC1 constituting more than a half of the ILC in both healthy skin and lesional HS skin. The numbers of ILC1 in the lesional skin were significantly higher than in healthy skin. In addition, the numbers of ILC1 were higher than numbers of ILC2 and ILC3 in the lesional HS skin, suggesting that ILC1 might contribute to the inflammation in the active lesion.

Analysis of blood samples indicates that TNF inhibition treatment affects the systemic numbers of ILC in HS patients. While HS patients had significantly lower numbers of ILC in the blood and increased numbers in the skin compared to healthy controls, there was a noticeable trend with anti-TNF treatment in restoring the numbers of ILC in the blood to levels characteristic of healthy individuals.

Given that the absolute numbers of ILC in HS patients were relatively reduced in the blood and increased in lesional skin, this suggests that ILC may traffic from the blood to the inflamed skin in HS and contribute to inflammation. Furthermore, one could speculate that ILC might play an early role in initiating inflammation in HS given the increased proportion of total ILC and ILC2 and ILC3 subsets in peri-lesional than in lesional skin. However, further investigation is needed to characterise the functional capacity of ILC to contribute to immune responses in HS and to improve the overall understanding of the pathogenesis of HS.

## Supporting information

S1 Fig Representative flow cytometric dot plots for ILC subsets in blood and skin of healthy individuals, HS patients and psoriasis patients. ILC were defined as live CD45^+^ cells, which were then gated on forward scatter height (FSC-H) vs FSC area (FSC-A) to exclude doublets. Singlets were then gated on side scatter area (SSC-A) vs FSC-A to select lymphocytes. ILC were then defined as CD127^dim^ lineage-negative, with the lineage ‘cocktail’ consisting of antibodies specific for CD1a, CD3, CD11c, CD14, CD19, CD34, CD94, CD123, CD303, FCεRI, TCRαβ and TCRγδ. ILC1 were then defined as CD117^-^CD161^+^, ILC2 as CD294^+^ and ILC3 as CD117^+^ NKP44^+/-^ (HC = healthy control, HS = hidradenitis suppurativa, PSO = psoriasis, NL = non-lesional, LES = lesional).
(TIF)
