# Supplementary figures and images for: Innate lymphoid cell (ILC) subsets are enriched in the skin of patients with hidradenitis suppurativa
Source: PLoS One. 2023 Feb 13;18(2):e0281688. doi: 10.1371/journal.pone.0281688 (PMC9924995; doi:10.1371/journal.pone.0281688)

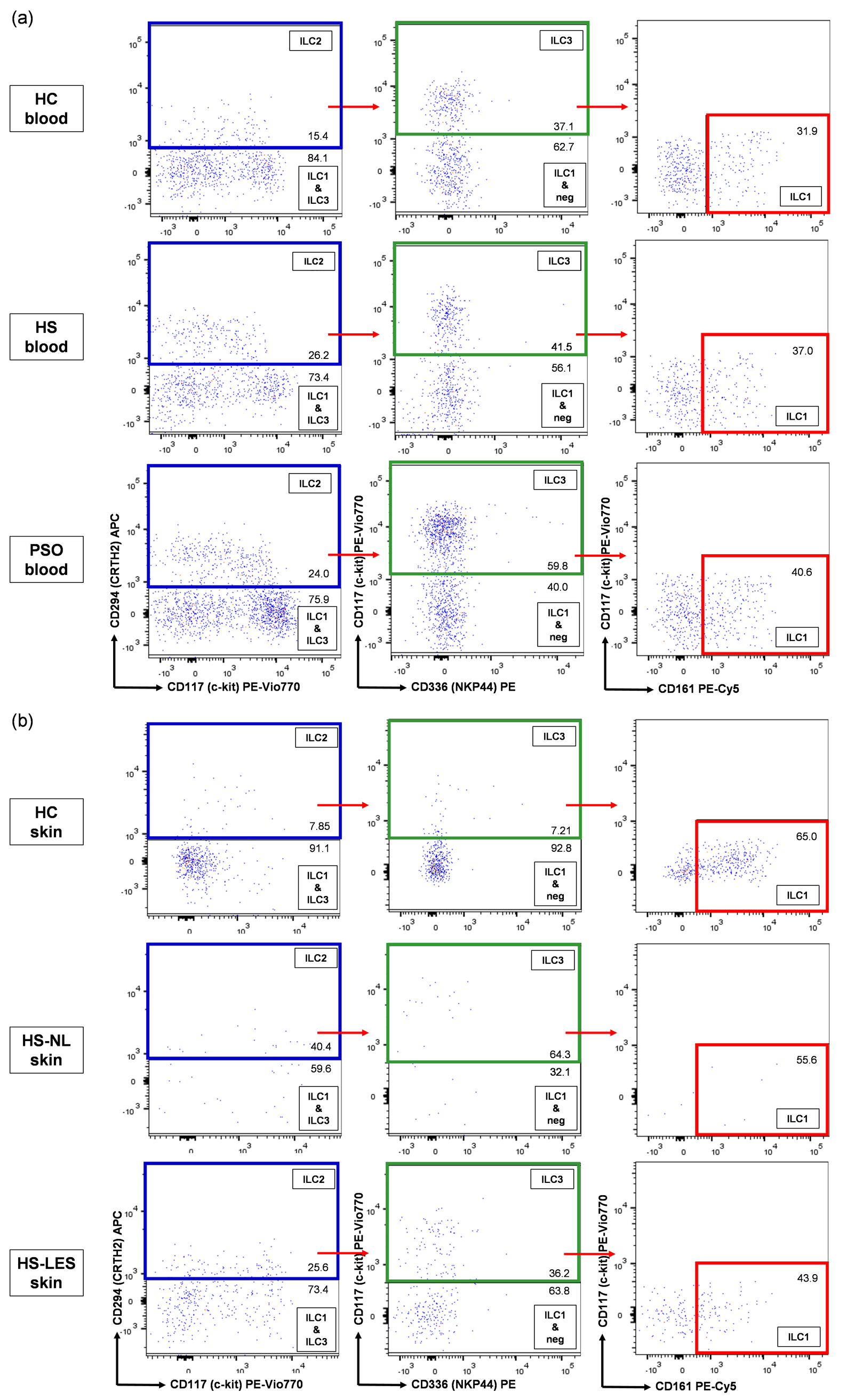

Supplement: S1 Fig — ILC were defined as live CD45+ cells, which were then gated on forward scatter height (FSC-H) vs FSC area (FSC-A) to exclude doublets. Singlets were then gated on side scatter area (SSC-A) vs FSC-A to select lymphocytes. ILC were then defined as CD127dim lineage-negative, with the lineage ‘cocktail’ consisting of antibodies specific for CD1a, CD3, CD11c, CD14, CD19, CD34, CD94, CD123, CD303, FCεRI, TCRαβ and TCRγδ. ILC1 were then defined as CD117-CD161+, ILC2 as CD294+ and ILC3 as CD117+ NKP44+/- (HC = healthy control, HS = hidradenitis suppurativa, PSO = psoriasis, NL = non-lesional, LES = lesional). (TIF) [file pone.0281688.s001.tif]
